# Protective Effect of Cyclically Pressurized Solid–Liquid Extraction Polyphenols from *Cagnulari* Grape Pomace on Oxidative Endothelial Cell Death

**DOI:** 10.3390/molecules23092105

**Published:** 2018-08-21

**Authors:** Anna Maria Posadino, Grazia Biosa, Hatem Zayed, Haissam Abou-Saleh, Annalisa Cossu, Gheyath K. Nasrallah, Roberta Giordo, Daniela Pagnozzi, Maria Cristina Porcu, Luca Pretti, Gianfranco Pintus

**Affiliations:** 1 Department of Biomedical Sciences, School of Medicine, University of Sassari, Viale San Pietro 43/B, 07100 Sassari, Italy; posadino@uniss.it (A.M.P.); cossuannalisa@libero.it (A.C.); 2 Porto Conte Ricerche S.r.l, Tramariglio, Alghero, 07041 Sassari, Italy; biosa@portocontericerche.it (G.B.); pagnozzi@portocontericerche.it (D.P.); cristina.porcu@ss.icb.cnr.it (M.C.P.); 3 Department of Biomedical Sciences, College of Health Sciences, Qatar University, P.O. Box 2713 Doha, Qatar; hatem.zayed@qu.edu.qa (H.Z.); gheyath.nasrallah@qu.edu.qa (G.K.N.); 4 Department of Biological and Environmental Sciences, College of Arts and Sciences, Qatar University, P.O. Box 2713 Doha, Qatar; hasaleh@qu.edu.qa; 5 Biomedical Research Center, Qatar University, P.O. Box 2713 Doha, Qatar; roberta.giordo@qu.edu.qa

**Keywords:** *Cagnulari* marc, Naviglio Extractor^®^, green extraction, endothelial cell, oxidative stress, polyphenols

## Abstract

The aim of this work is the evaluation of a green extraction technology to exploit winery waste byproducts. Specifically, a solid–liquid extraction technology (Naviglio Extractor^®^) was used to obtain polyphenolic antioxidants from the *Cagnulari* grape marc. The extract was then chemically characterized by spectrophotometric analysis, high-performance liquid chromatography, and mass spectrometry, revealing a total polyphenol content of 4.00 g/L ± 0.05, and the presence of anthocyanins, one of the most representative groups among the total polyphenols in grapes. To investigate potential biological activities of the extract, its ability to counteract hydrogen peroxide-induced oxidative stress and cell death was assessed in primary human endothelial cells. The 3-(4,5-dimethylthiazol-2-yl)-2,5-diphenyltetrazolium bromide (MTT) test, used to assess potential extract cytotoxicity, failed to show any deleterious effect on cultured cells. Fluorescence measurements, attained with the intracellular reactive oxygen species (ROS) probe 2′,7′-dichlorodihydrofluorescein diacetate (H_2_DCF-DA), revealed a strong antioxidant potential of the marc extract on the used cells, as indicated by the inhibition of the hydrogen peroxide-induced ROS generation and the counteraction of the oxidative-induced cell death. Our results indicate the Naviglio extraction, as a green technology process, can be used to exploit wine waste to obtain antioxidants which can be used to produce enriched foods and nutraceuticals high in antioxidants.

## 1. Introduction

Although wine production is one of the most important agricultural activities worldwide [1], the problems related to its waste treatment or disposal are far from being resolved [2]. Contamination problems related to winery waste byproducts have been raised [3,4]. Indeed, wine waste, such as lees and grape marc, may exert phytotoxic effects if applied to crops [4] or wetlands [5], since their content of specifc micronutrients and heavy metals is incompatible with the agricultural requirements [4,5,6]. Nevertheless, most of these byproducts can be used for different purposes, including the production of functional foods, dietary supplements, cosmetics, and pharmaceuticals [7,8]. The wine byproducts represent a considerable burden as industrial waste, due to the presence of variable phenolic contents in the wine, depending on the type of grape, the part of the tissue, and the processing conditions [4]. However, this burden can be significantly reduced using new or modified processing methods that aim to generate useful wine bioproducts, such as natural antioxidants. In this context, phenolic compounds with high antioxidants properties have been extracted from grape marc using new extraction techniques, such as pressurized liquid extraction and supercritical carbon dioxide [9].

Extraction methods, such as supercritical fluid extraction (SFE), pressurized liquid extraction (PLE), and assisted microwave irradiation extraction (MAE), can be employed as an alternative to conventional extraction techniques, since they have many practical aspects, including the reduction of solvent consumption and increasing the extraction rate process [9,10]. In this context, the Naviglio Extractor^®^ is a solid–liquid extraction technology that works by generating a negative gradient pressure from the inside toward the outside of the solid matrix, which transports the extractable material outside of the matrix, by causing a suction effect. This extraction technique has been proven to be reproducible, quantitatively comparable, and affordable compared to other techniques, such as SFE and accelerated solvent extraction (ASE) [11,12,13,14]. Although the Naviglio Extractor^®^ has been employed on several matrices [13,15,16,17], to our knowledge, its use on grape marc is yet to be explored. In addition, to be valuable, an extraction technology should not affect or should be able to retain the biological activities of the compounds extracted from that matrix, and therefore, its employment needs to be tested for this aspect.

Association of increased reactive oxygen species (ROS) with cardiovascular diseases (CVD) [18] suggests that counteracting oxidative stress with antioxidants could prevent disease occurrence or ameliorate their effects. For this reason, a significant amount of attention is now focusing on naturally occurring antioxidants as potential candidates for CVD prevention and/or treatment. The endothelial cell (EC) plays a crucial role in the integration and modulation of signals within the vascular wall [19], and perturbation of such homeostasis by oxidative damage is the trigger for the onset and development of CVD [18]. Therefore, we used human umbilical vein endothelial cells (HUVECs) to evaluate the potential antioxidant activity of the obtained extract. Indeed, HUVECs have been reported to be a useful model to study the vascular cells’ response to natural antioxidant treatment [20,21,22,23,24,25].

Thus, the aim of the present work is to investigate whether (i) the green technology, Naviglio Extractor^®^, can be used to extract valuable antioxidant products from the wine waste, and (ii) the obtained products exert antioxidant activity, by protecting human endothelial cells against H_2_O_2_-induced oxidative stress and cell death.

## 2. Materials and Methods

### 2.1. Chemicals

Unless stated in the text, all the used reagents were from Sigma-Aldrich (St. Louis, MO, USA).

### 2.2. Pomace Acquisition and Preparation

The pomace of *Vitis vinifera* var. *Cagnulari* was purchased from a winery cooperative (Santa Maria La Palma, Alghero, Italy). Around 50 kg of exhausted pomace (RH% 52.6 ± 2.1) was air-dried in a cabinet tray drier with air flowing in alternate directions at 35 °C. After 72 h, the dried pomace (RH% 5.76 ± 0.2) was milled and sieved to a particle size of less than 4 mm diameter, and then vacuum stored at 4 °C until use.

### 2.3. Pomace Extraction Procedure

The detailed principle and procedures for the Naviglio Extractor have been previously reported [11]. Briefly, the extractor works by alternating a static phase with a dynamic one; during the static phase, the extracting solvent is brought to a pressure of about 10 atm, and is kept in this state for sufficient time to reach an equilibrium between the inside and the outside pressure of the solid matrix; at the end of the static phase, a dynamic phase begins, produced by a sudden movement of the pistons, which generates a rapid pressure decrease. The negative pressure gradient created between the inside and the outside of the solid matrix allows the extraction, due to a suction effect [11]. About 4 kg of dried pomace was recovered in a porous bag made of 50 µm filter membrane, and then introduced into the extraction chamber of the Naviglio Extractor (Mod. EXNA 1015) in 12.2 kg of water/ethanol 60:40 (*v*/*v*). To allow total polyphenols recovery, 21 extractive cycles of 1 min and 25 s each for a total of 38 min (12 min in static phase and 26 min in dynamic phase) were performed. The obtained hydro-alcoholic extract was recovered and stored at −20 °C.

### 2.4. Chemical Characterization of the Extract

#### 2.4.1. Folin–Ciocalteu (FC) Assay

The total polyphenol content of the extract was determined spectrophotometrically according to the classical Folin–Ciocalteau (FC) method. A 50 μL aliquot of standard solution, composed by a set of gallic acid solutions (concentrations from 50 to 500 ppm), was added to different quartz cuvettes (1 cm optical path) filled with 2350 μL of water and 150 μL of FC reagent. After 3 min, a solution of 20% sodium carbonate (450 μL) was added. Blank solution was prepared in the same way, but adding water instead of samples. All solutions were mixed in the dark for 2 h at RT, and then the absorbance was recorded at 760 nm with a Varian Cary 3E UV–vis spectrophotometer. Before analysis, 20 μL of extract were desalted and purified through a passage in a solid phase extraction C18 cartridges (Phenomenex, Torrance, California, USA, 55 μm, 70 A) equilibrated with 10% HCOOH solution. Then the extract was diluted 50 times, then treated with the same procedure used for gallic standards. The total polyphenol concentration was estimated by linear interpolation with a calibration curve made by the set of gallic acid solutions.

#### 2.4.2. High-Performance Liquid Chromatography Analysis

Qualitative analysis of the extract was performed by high-performance liquid chromatography (HPLC) using an HP 1100/1200 instrument (Agilent Technologies, Palo Alto, CA, USA), equipped with an autosampler (100 µL sample loop), and a diode array detector [26]. About 225 μg of raw extract was loaded in 100 µL of HPLC starting conditions (6% of eluent A, composed by water/acetonitrile/formic acid, 87:3:10 *v*/*v*/*v*) on a C12 column (Synergi 4 µm Max-RP 80 A, 250 × 4.6 mm ID, Phenomenex, Torrance, California, USA), preceded by a Synergi guard column (4 × 3.00 mm, Phenomenex) with the same stationary phase, at room temperature. The flow rate was set at 0.5 mL/min with a multistep gradient of eluent B (water/acetonitrile/formic acid, 40:50:10 *v*/*v*/*v* in A from 6% to 90%, according to the scheme provided in Table 1. Elution was followed by recording the absorbance between 190 and 700 nm. As a reference, a standard pool composed by 60 µL of a 2.5 ng/µL solution of malvidin-3-*O*-glucoside chloride, 60 µL of a 5 ng/µL solution of cyanidin-3-*O*-glucoside chloride, and 60 µL of a 2.5 ng/µL solution of peonidin-3-*O*-glucoside chloride (Sigma-Aldrich) was run on the same gradient.

#### 2.4.3. Mass Spectrometry Analysis

Identification of anthocyanins was confirmed by mass spectrometry (MS). Raw extract sample and selected fractions collected from HPLC run were analyzed using a MALDI-TOF Micro MX (Waters, Manchester, UK) spectrometer [27], equipped with a nitrogen laser (337 nm), and reflectron analyzer, in delayed extraction mode. Samples were mixed with an equal volume of the matrix, which was a DHB solution (2,5-dihydroxybenzoic acid, 20 mg/mL in 90% acetonitrile). Spectra were acquired in the 100–800 mass to charge ratio (*m*/*z*). The *m*/*z* range was externally calibrated by acquiring the spectrum of a mixture of compounds with known masses. The calibration was performed by air-drying on the plate 2 µL of a 1:1 mixture of standard polyphenols (resveratrol, 228.0786 g/mol, 0.14 µg/µL, peonidin-3-*O*-glucoside chloride, 463.1240 g/mol, 0.28 µg/µL cyanidin-3-*O*-glucoside chloride, 449.3848 g/mol, 0.28 µg/µL, malvidin-3-*O*-glucoside chloride, 493.1346 g/mol, 0.28 µg/µL), and DHB solution.

### 2.5. Assessment of Extract Biological Activity

#### 2.5.1. Endothelial Cell Culture and Treatments

Human umbilical vein endothelial cells (HUVECs) were isolated from the vein of human umbilical cords and cultured as previously described [22,23,24,25,26,27,28]. Briefly, cells were detached from the interior of the umbilical vein of a 30 cm segment cord by treatment for 10 min at 37 °C with 0.05% (*w*/*v*) collagenase type II from *Clostridium hystolyticum* in medium M199 containing 100 U/mL of penicillin G sodium salt and 100 µg/mL streptomycin sulphate. HUVECs were harvested at 1000*g* for 10 min and finally resuspended in 5 mL medium M199 supplemented with 10% (*v*/*v*) fetal calf serum (FCS), 10% (*v*/*v*) newborn calf serum, 2 mM glutamine, and antibiotics. Cells were then plated in 25 cm^2^ tissue culture flasks (Falcon, Oxnard, CA, USA) and cultured in an atmosphere of 5% CO_2_/95% air. Cells were identified as endothelial cells by their “cobblestone” morphology and by factor VIII staining. Second passage HUVECs coming from a pool of different umbilical cords were used for experimentation. The day before each experiment, cells were plated in 48-well plates (Corning, Lowell, MA, USA) at a concentration of 10^4^ cells/mL, and pretreated with the extract for 3 h, before oxidative stress was induced for 2 h by treatment with the indicated concentration of hydrogen peroxide (H_2_O_2_). In agreement with our previous study using a supercritical fluid extraction (SFE) extract of *Salvia desoleana* (*S. desoleana*) [25], the doses of 0.1, 1, and 10 µg/mL were tested in our human vascular model.

#### 2.5.2. Measurements of Intracellular ROS

Intracellular ROS levels were determined by using the ROS molecular probe 2′,7′-dichlorodihydrofluorescein diacetate (H_2_DCF-DA) (Molecular Probe, Eugene, OR, USA), as previously described [23,24]. Within the cell, esterases cleave the acetate groups on H_2_DCF-DA, thus trapping the reduced form of the probe (H_2_DCF). Intracellular ROS oxidize H_2_DCF, yielding the fluorescent product, DCF. Cells were incubated for 3 h with the concentrations of extract indicated in the figure legends. After treatment, cells were incubated for 30 min with Hanks’ Balanced Salt Solution (HBSS) containing 5 µM H_2_DCF-DA, then washed twice with HBSS and assessed for the fluorescence by using a Tecan GENios plus microplate reader (Tecan, Männedorf, CH, USA) in a light-protected condition. Treatment-induced variation of fluorescence was measured for 2 h in cell culture medium without phenol red. Excitation and emission wavelengths used for fluorescence quantification were 485 nm and 535 nm, respectively. All fluorescence measurements were corrected for background fluorescence and protein concentration. Using untreated cells as a reference, the overall anti- and pro-oxidant outcome was evaluated by comparison of five different measurements and expressed as percent of controls.

#### 2.5.3. Assessment of Cell Viability

Potential toxicity and potential protection against oxidative cell death of the obtained extract were investigated under the different experimental conditions. To this end, cell viability was assessed in 96-well plates (BD Falcon) by using the colorimetric 3-(4,5-dimethylthiazol-2-yl)-2,5-diphenyltetrazolium bromide (MTT) assay, as previously reported [21,29]. To determinate potential toxicity, cells were treated for 20 h with the indicated concentrations of extract. To determinate potential protection against oxidative-induced cell damage, cells were pre-incubated for 3 h with the concentrations of extract indicated in the figure legends, then H_2_O_2_ (75 µM) was added for 20 h. After treatments, cells were added with 20 µL MTT solution (5 mg/mL) in cell culture medium and incubated at 37 °C in a cell incubator for additional 4 h; the medium was then removed, and the cell monolayer was washed twice with HBSS. The converted dye was solubilized with acidic isopropanol (0.04 N HCl in absolute isopropanol), and plates were analyzed at 570 nm using a GENios Plus microplate reader (Tecan, Männedorf, CH, USA) with background subtraction at 650 nm. All the measurements were corrected for protein concentrations and results expressed as a percentage of untreated controls.

### 2.6. Statistical Analysis

Data were checked for normal distribution and processed by one-way analysis of variance (ANOVA) followed by post hoc Newman–Keuls multiple comparison tests to determine the differences between mean values among treatments, with significance defined as *p* < 0.05. All the analyses were performed using the GraphPad Prism 6 software (GraphPad Software Inc., San Diego, CA, USA).

## 3. Results and Discussion

The treatment and disposal of waste originating from agricultural activities are important issues that are far from being resolved [2,4,5,6]. Fortunately, fruit and vegetable processing waste is rich in valuable compounds, such as antioxidants, and their utilization as a source of natural food additives is gaining a great deal of attention [7,8]. On the other hand, the pressing requirement to resolve food waste problems in an eco-sustainable manner necessitates the resolution of disadvantages associated with old extraction methods and promoting waste exploitation by employing green technologies. In this context, the rapid solid–liquid dynamic Naviglio^®^ extractor represents a technology of solid–liquid extraction that possess several advantages as compared to the other currently existing extractive techniques. Contrary to many of the current methods, which aim to heat the extractive system to raise efficiency and shorten extraction times, the Naviglio^®^ extractor carries out the extraction at room or sub-room temperature, and uses an increase of pressure of the extracting liquid on the solid matrix, thus avoiding thermal stress on thermolabile substances [11]. Moreover, the employment of higher pressures allows a reduction in the extraction time and a concomitant improvement of the extraction process [11,12,13,14].

**Figure 1:**
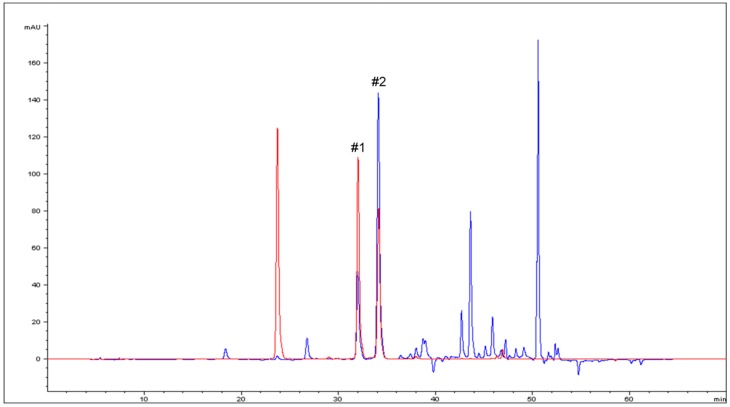
HPLC analysis. Overlapping of HPLC chromatograms obtained by anthocyanin standards (red curve) and pomace extract (blue curve), wavelength = 520 nm. #1 and #2 indicate the fractions collected and further analyzed by MS.

**Figure 2:**
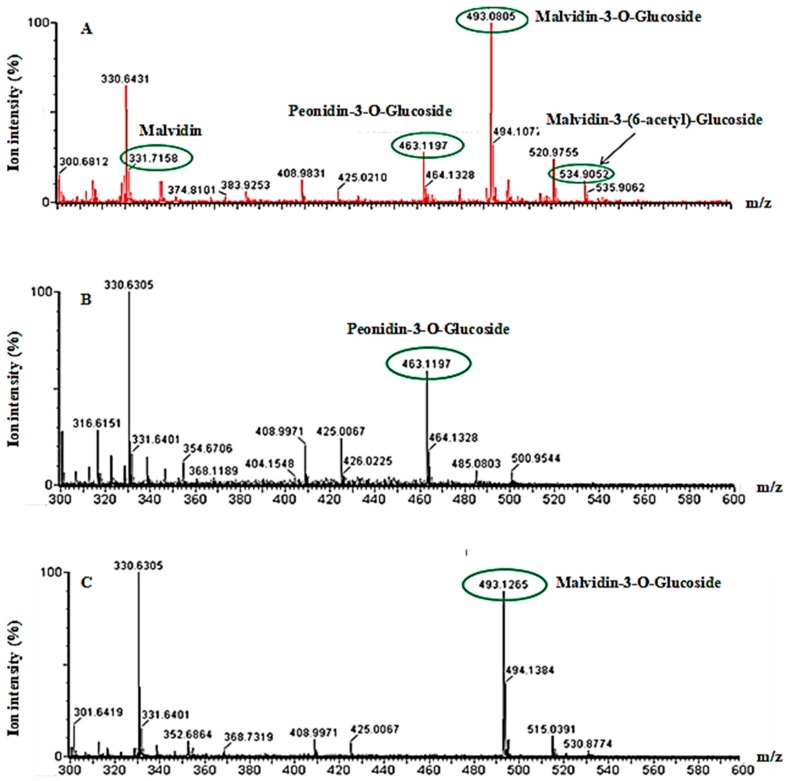
MALDI-TOF MS analysis. (**A**) Raw extract spectrum, confirming the presence of several anthocyanins, including malvidin, malvidin-3-*O*-glucoside, peodinin-3-*O*-glucoside, and malvidin-3-(6-acetyl)-glucoside. (**B**) Fraction 1 and (**C**) Fraction 2 spectra, confirming the presence of peodinin-3-*O*-glucoside and malvidin-3-*O*-glucoside, respectively.

In this study, we used the Naviglio^®^ extractor on *Cagnulari* grape marc to isolate valuable natural antioxidants from wine waste. Indeed, while wine processing residues are unusable for agricultural purposes, its content in polyphenol antioxidants constitutes one of the higher value options for wine byproducts exploitation, since they provide many health benefits [7,8]. As revealed by the FC assay performed on the extract, the Naviglio^®^ extractor was able to recover 4.00 g/L ± 0.05 of total polyphenol. This finding indicates that valuable polyphenolic substances are still present in the wine waste, and that the green technology employed was able to extract them from the waste matrix. As compared to other extraction technologies, our data indicate that the Naviglio^®^ extractor recovered a number of polyphenols, characteristic of the used matrix [30,31]. Indeed, the overlap of pomace extract (blue curve) and standard (red curve) chromatograms clearly shows the correspondence between two anthocyanin standard peaks and two species present in the extract (#1 and #2, Figure 1). MS analysis of the pre-HPLC extract confirmed the presence of several anthocyanins characteristics of the exploited matrix, including malvidin, peonidin-3-*O*-glucoside, malvidin-3-(6-acetyl)-glucoside, and M-3-G (Figure 2A). Moreover, the MS analysis of HPLC fraction 1 (#1) and 2 (#2) further confirmed the presence of the specific anthocyanins peonidin-3-*O*-glucoside and M-3-G respectively (Figure 2B,C). Interestingly, the extract chromatogram (Figure 1, blue curve) showed a considerably high abundance of malvidin-3-glucoside (#2), in agreement with previous findings [30,31,32].

Within the different natural antioxidants, plant polyphenols are considered among the most important providers of health benefits, such as those related to the cardiovascular system [33]. In this context, red wine polyphenols are thought to be responsible for the cardiovascular benefits associated with the regular consumption of moderate amounts of wine [34,35]. M-3-G is one of the anthocyanins present in red wine responsible for the red pigmentation of red grapes and red wine [32]. While its antioxidant activity in vitro has been reported [36], as compared to the resveratrol, a natural red wine polyphenol, relatively few reports investigated its antioxidant activity and vasculoprotective effect in a vascular cell model. Indeed, although chemical tests to assess antioxidant activity are often used, the best approach to study the antioxidant activity/effect of a given compound would be directly in vivo or in a cellular model. An in vivo model, such as the cell, would provide the optimal environment for possible interaction (e.g., compound–cellular receptor, compound–cellular signal transduction) that would be missed in a chemical assay system.

**Figure 3:**
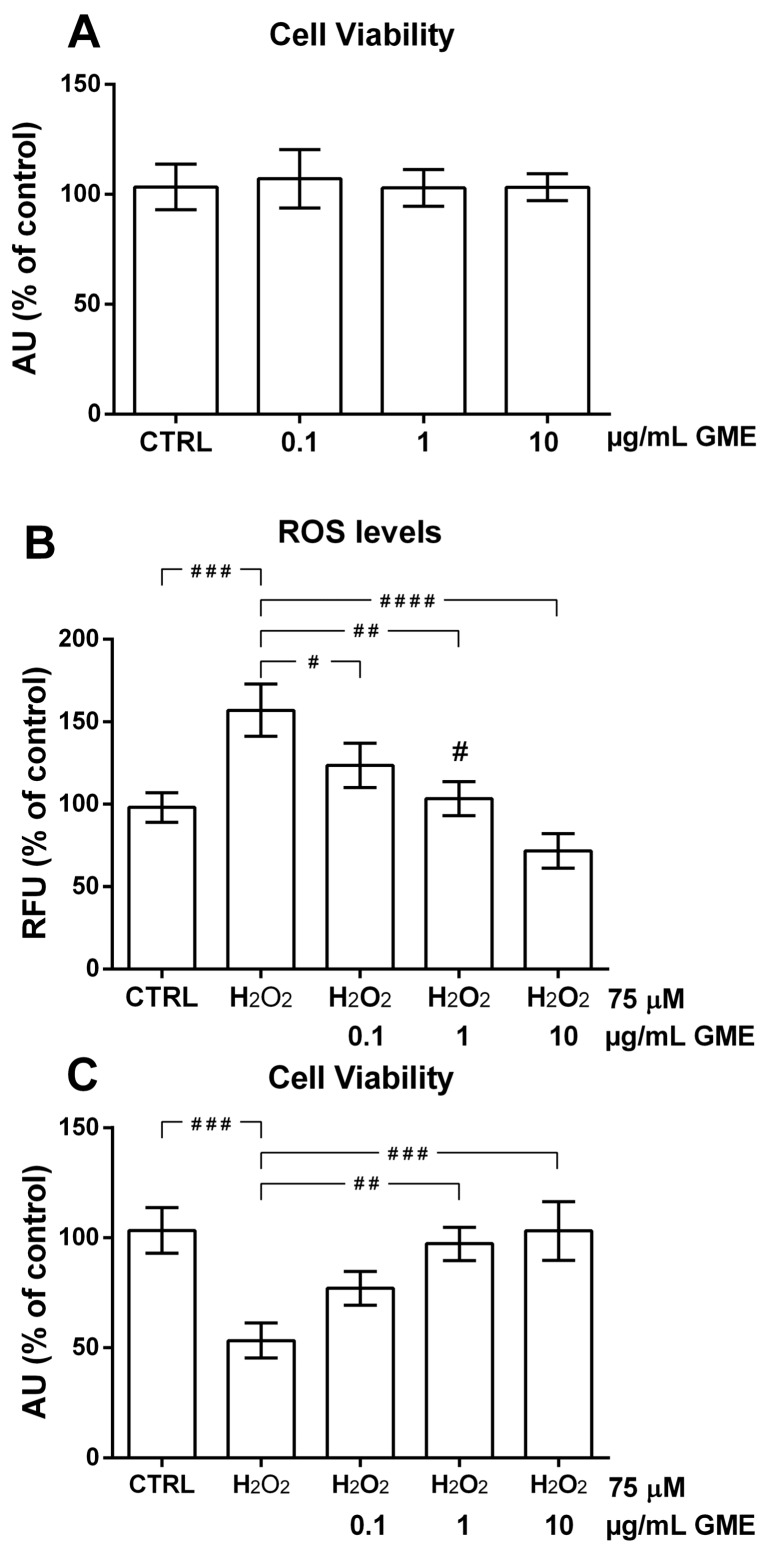
Effect of grape marc extracts on human umbilical vein endothelial cell (HUVEC) viability. (**A**) HUVECs were incubated for 20 h with the indicated concentrations of the extract, then cell viability was determined using the MTT assay, as reported in the materials and methods. Grape mark extract inhibits hydrogen peroxide (H_2_O_2_)-induced ROS generation in HUVECs. (**B**) HUVECs were exposed for 3 h to the indicated concentrations of grape marc extracts, and then incubated in the absence (CTRL) or presence of 75 µM H_2_O_2_. Fluorescence was measured, as reported, in the Materials and Methods. #, *p* = 0.0428; ##, *p* = 0.0021; ###, *p* = 0.001; ####, *p* = 0.0001. Grape mark extract inhibits hydrogen peroxide (H_2_O_2_)-induced oxidative cell death of HUVECs. (**C**) HUVECs were exposed for 3 h to the indicated concentrations of grape marc extracts and then incubate for 20 h in the absence (CTRL) or presence of 75 µM H_2_O_2_. Cell viability was assessed as reported in the materials and methods. Data are expressed as percentage of the control. CTRL, untreated cells; H_2_O_2_, hydrogen peroxide; GME, Grape marc extract. ##, *p* = 0.0017; ###, *p* = 0.0006.

The endothelium plays a pivotal role in cardiovascular homeostasis, and oxidative-induced endothelial cell (EC) injury is the key step in the onset and progression of CVD [18]. Therefore, the EC is a very useful model to investigate the potential effects of M-3-G on the vascular system. When tested on our cell vascular model, the extract obtained from the *Cagnulari* grape marc with the Naviglio Extractor^®^ was safe. In fact, based on our previous experimentations with extracts from *Salvia desoleana* [25], we tested the pomace extract at concentrations of 0.1, 1, and 10 µg/mL without evidence of toxic effects on cultured cells (Figure 3A). Based on the high polyphenol content found in the grape marc extract, we sought to investigate whether it may counteract H_2_O_2_-elicited oxidative changes in our vascular models. Therefore, we pre-incubated the cells with the three different concentrations of the extract, and then we cultured them in the presence and absence of H_2_O_2_ to assess a potential antioxidant effect. Using the ROS fluorescent sensor H_2_DCF-DA, the data derived from five pooled measurements were expressed as percentages of the untreated cells (CTRL) in Figure 3B. Exposure of the HUVEC cells to these increasing concentrations of the extract showed significant antioxidant effects with respect to the H_2_O_2_-treated cells (Figure 3B). Since oxidative stress is known to induce cell damage and even death, we sought to determine whether the observed antioxidant effect could be protective against the H_2_O_2_-induced oxidative damage. To this end, we pre-treated the cell with the indicated concentrations of extract and then we measured the cell viability by using the MTT assay both in oxidatively-stressed and -unstressed cells. Consistently with the observed antioxidant effect on H_2_O_2_-induced ROS generation, pre-treatment of cells with increasing doses of grape marc extract was able to significantly protect HUVECs from the oxidative cell death elicited by H_2_O_2_ (Figure 3C).

Due to the ever-growing production of waste by modern society, the environmental sustainability requirements related to waste treatment or disposal are becoming an issue of primary importance [3,4]. In this context, exploiting these residues to obtain valuable compounds by mean of green technologies may be the answer to this problem, both in terms of environmental sustainability and for potential profit. In some case, these residues can indeed be an alternative source for obtaining natural antioxidants, which are considered safer in comparison with synthetic antioxidants, largely used in the food industry with undesirable effects on the enzymes of human organs [7,8,37,38]. Hence, phenolic compounds can be considered added-value byproducts, which justifies their isolation from the industrial wastes [38]. Antioxidant and anti-inflammatory activities of anthocyanins have been extensively reported, therefore promoting studies concerning their extracts from different sources [4,39]. In this study, we used a green extraction technology (Naviglio^®^) to provide a proof of principle that wine industry wastes can be a rich resource of natural antioxidants.

As reported in the current study, the employed extraction technology was able to extract a remarkable amount (4.00 g/L ± 0.05) of phenolic compounds from the wine waste (*Cagnulari* Grape Marc). The performed chemical analysis revealed the Naviglio Extractor was able to recover specific anthocyanins, such as the malvidin, peonidin-3-*O*-glucoside, malvidin-3-(6-acetyl)-glucoside, and M-3-G, which are characteristic phenolic compounds of the exploited matrix. Tested on a cell vascular model, the Naviglio extract showed a strong antioxidant effect. Indeed, in H_2_O_2_-treated cells exposed to a low concentration of extract (1 µg/mL) the levels of ROS did not significantly differ from the one in control cells (CTRL), showing 53% more antioxidant effect as compared to cells treated with only H_2_O_2_ (*p* = 0.0021) (Figure 3B). At higher concentration (10 µg/mL), the extract showed an antioxidant effect that was 85% higher when compared to cells treated with only H_2_O_2_ (*p* = 0.0001). The observed dose-dependent antioxidant was paralleled to a protective effect of similar extent when cells were analyzed for H_2_O_2_-induced cell death in the presence of the different extract concentrations (Figure 3C). Noteworthy, contrary to what was observed for other natural antioxidants, such as resveratrol and coumaric acid for instance [22,23,24], this powerful antioxidant effect was free of any cytotoxicity or harmful effects on cells, even when treated with increasing concentrations of extract (Figure 3A), meaning that the extract does not affect the levels of intracellular ROS needed to finely control the cellular functions.

## 4. Conclusions

Our results show that the grape pomace can be an important source of polyphenolic substances that provide antioxidant activity and vasculoprotective effect. The Naviglio^®^ is a green extractive technology that does not make use of any solvent or thermal treatments, and is affordable compared to other conventional solvents or supercritical fluid phase extraction procedures. Our current data indicate that the green technology used to exploit the wine waste is capable of extracting antioxidants compounds that are characteristic of the employed grape pomace, namely anthocyanins. Considering that red grape is used in millions of tons in wine-producing countries, the polyphenolic compounds extracted from grape processing byproducts such as grape marc, seeds, or peels could be used as sources of natural antioxidants to be employed in different contexts. Ultimately, besides the “green aspect” related to the technology employed and the waste recycling, our proposed extraction process may be applied to other natural sources of polyphenols in various fruits, spices, and dried herbs, such as cocoa products, some berries, flaxseeds, and nuts (chestnut, hazelnut), and some vegetables, including olive and artichoke [40], which play an important role on cardiovascular protection. From this perspective, further studies need to be performed to investigate other polyphenol-rich byproducts, and to highlight their antioxidant and protective role in health and disease.

## Figures and Tables

**Table 1:** HPLC gradient used for the anthocyanin compounds separation.

Time (min)	Eluent B (%)
0	6
5	6
25	20
40	40
45	60
50	90
60	90
